# Prediction of Freezing of Gait in Parkinson’s Disease Using a Random Forest Model Based on an Orthogonal Experimental Design: A Pilot Study

**DOI:** 10.3389/fnhum.2021.636414

**Published:** 2021-03-22

**Authors:** Zhonelue Chen, Gen Li, Chao Gao, Yuyan Tan, Jun Liu, Jin Zhao, Yun Ling, Xiaoliu Yu, Kang Ren, Shengdi Chen

**Affiliations:** ^1^Gyenno Science Co., Ltd., Shenzhen, China; ^2^Department of Neurology, Ruijin Hospital Affiliated to Shanghai Jiao Tong University School of Medicine and Center for Excellence in Brain Science and Intelligence Technology, Chinese Academy of Sciences, Shanghai, China; ^3^School of Artificial Intelligence and Automation, Huazhong University of Science and Technology, Wuhan, China

**Keywords:** orthogonal experimental design, optimization, freezing of gait, Parkinson’s disease, fog prediction

## Abstract

**Purpose:**

The purpose of this study was to introduce an orthogonal experimental design (OED) to improve the efficiency of building and optimizing models for freezing of gait (FOG) prediction.

**Methods:**

A random forest (RF) model was developed to predict FOG by using acceleration signals and angular velocity signals to recognize possible precursor signs of FOG (preFOG). An OED was introduced to optimize the feature extraction parameters.

**Results:**

The main effects and interaction among the feature extraction hyperparameters were analyzed. The false-positive rate, hit rate, and mean prediction time (MPT) were 27%, 68%, and 2.99 s, respectively.

**Conclusion:**

The OED was an effective method for analyzing the main effects and interactions among the feature extraction parameters. It was also beneficial for optimizing the feature extraction parameters of the FOG prediction model.

## Highlights

- A novel method was developed to predict FOG in PD.
- An OED was first used to obtain the optimal feature extraction parameters.
- The main effects of the feature extraction parameters were analyzed first.
- Interactions among the feature extraction parameters were analyzed.

## Introduction

Parkinson’s disease (PD) is a neurodegenerative disease characterized by the degeneration of dopaminergic neurons of the substantia nigra resulting in bradykinesia, rigidity, tremor, and postural instability (Revuelta et al., 2015; Li et al., 2019). Over 50% of the PD patients who have lived with the disease for more than 10 years are affected by freezing of gait (FOG) (Okuma, 2006). In addition, FOG may occur in 25% of patients with early PD (Moore et al., 2008). FOG is defined as not being able to start or continue walking and feeling as if the feet have been “glued” or “magnetized” to the ground (Handojoseno et al., 2012). This condition causes both physical and psychological distress. Approximately 60% of PD patients experience falls each year, and they also have to endure multiple falls caused by complications and fall-related injuries (Wood et al., 2002; Moore et al., 2008; Allcock et al., 2009; Latt et al., 2009; Contreras and Grandas, 2012).

The response of FOG in PD to pharmaceutical treatment is limited (Okuma, 2006). However, previous works have shown that cueing-based training has specific effects on gait, freezing, and balance (Lim et al., 2005). Many works have aimed to develop small wearable devices that can detect FOG episodes (Han et al., 2003; Moore et al., 2008, 2013; Bachlin et al., 2009; Jovanov et al., 2009; Mazilu et al., 2012; Pepa et al., 2015; Zach et al., 2015). Gait recognition is a gait assessment tool that uses a machine learning (ML) algorithm. Researchers have attempted to develop a wearable system to improve the accuracy of identifying FOG events. To optimize the system, multiple aspects were investigated, including different sensors and their locations, a subset of the features extracted, different gait recognition models, and hyperparameters. Bächlin et al. (2009) used a threshold algorithm based on the freezing index and signal power to recognize FOG. Their model was more sensitive when sensors were worn on the hips. Mazilu et al. (2012) showed that the mean latency of FOG detection increased linearly with the window size, but the classification performance increased quickly and then stabilized. Using an ML algorithm such as random forest (RF), a single sensor was sufficient for FOG detection, and the placement of this sensor had little effect on the detection. Ensemble methods such as boosting and bagging are more appropriate than other methods (Mazilu et al., 2012). Mikos et al. (2017) found that features maximized their mutual information (MI) at different window lengths. The efficient parameter optimization method in gait recognition has not been fully studied. We found that when the dataset is very large, optimizing the hyperparameters is a very time-consuming process, so an efficient parameter optimization method needs to be discussed. Some works have studied the effects of feature extraction parameters, while the interaction among parameters has not received enough attention. We propose a statistical experimental design, i.e., an orthogonal experimental design (OED), to optimize the hyperparameters and analyzes the interaction.

Furthermore, by utilizing such a wearable system, timely rhythmic cues can be provided after FOG is detected. These systems have advantages in shortening the duration of FOG and, by providing immediate (at least hundreds of milliseconds) rhythmic cues as a response to the FOG signal, will further improve these systems. Therefore, FOG prediction is necessary to solve the response problem and further avoid intervention failure. An unsupervised feature learning decision tree can be used to perform FOG prediction (Mazilu et al., 2013). Mazilu et al. (2015b) explored the association of gait with electrocardiogram (ECG) and skin-conductance response (SCR) features and then fit the data to a multivariate Gaussian distribution, which can be used for FOG detection. In previous studies, parameters such as the window size and the defined duration of precursor signs of FOG (preFOG; all the references to preFOG in this article mean preFOG in PD) are based on experience (Mazilu et al., 2013, 2015b). However, the principles of objectification, multipurpose use, and simplification (OMS) have been the trend in the development of a novel behavioral assessment for PD (Asakawa et al., 2016a, b, 2019).

In this study, an RF model evaluated with an episode-based strategy was developed to predict upcoming FOG. This model can be adopted by wearable devices to activate an early intervention to avoid some of the FOG episodes.

## Materials and Methods

### Methodology Overview

**FIGURE 1:**
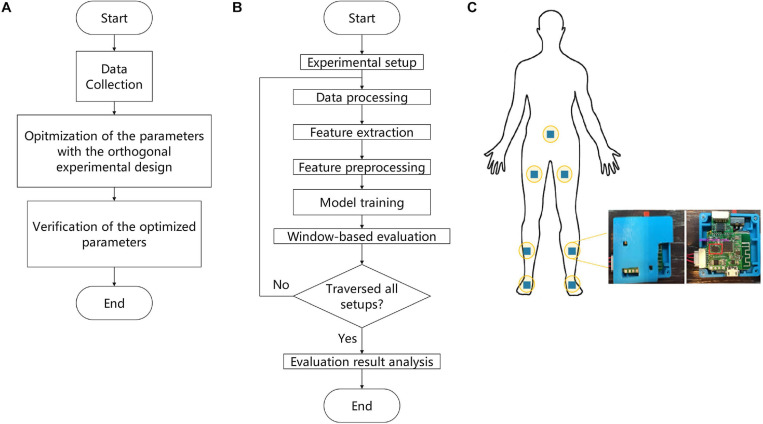
Flowchart of the proposed methodology. **(A)** Main experimental workflow. **(B)** Details of the parameter optimization process by means of the orthogonal experimental design (OED). **(C)** Schematic overview of the positions of the sensors.

The proposed methodology for building a model to predict FOG based on OED consists of three parts, as shown in Figure 1A: (a) data collection, (b) optimization of the parameters with the OED, and (c) verification of the optimized parameters. This article was focused on optimizing feature extraction parameters with the OED, and the details included seven steps, as shown in Figure 1B: (a) experimental setup, (b) data processing, (c) feature extraction, (d) feature preprocessing, (e) model training, (f) window-based evaluation, and (g) evaluation results analysis.

### Data Collection

The study participants were diagnosed with PD by movement disorder specialists according to the Movement Disorder Society (MDS) diagnostic criteria (Postuma et al., 2015). The eligibility criteria for the participants were a Hoehn–Yahr stage between 2 and 3 in FOG OFF state, not having cognitive dysfunction according to the Mini-Mental State Examination (MMSE), not having serious vision or hearing impairment, and not having any disease affecting walking ability. Participants were excluded if they had secondary PD causes, such as inflammatory, drug-induced, vascular, and toxin-induced Parkinsonism. Participants with other neurodegenerative diseases, such as progressive supranuclear palsy and multiple system atrophy, were also excluded. All the participants were familiar with the process and signed consent forms. Patients with severe PD (Hoehn–Yahr stage > 3.0) were not included due to safety issues. The characteristics of the patients are presented in Table 1. This study was approved by the ethics committee of the Ruijin Hospital, affiliated with the Shanghai Jiao Tong University School of Medicine.

**TABLE 1:** Characteristics of the included patients.

	Male	Female	*p*-value
Number (%)	8 (57.14)	6 (42.86)	–
Age, years, mean (SD)	71.83 (11.67)	69.20 (5.89)	0.642
Age of Onset, years, mean (SD)	5.00 (2.61)	6.40 (4.34)	0.549
Hoehn–Yahr Stage, *N* (%) 2 2.5 3	3 (37.50) 3 (37.50) 2 (25.00)	2 (33.33) 2 (33.33) 2 (33.33)	1.000
MDS–UPDRS score, mean (SD)	51.17 (5.56)	52.00 (7.81)	0.847
FOGQ score, mean (SD)	8.83 (1.17)	8.80 (0.84)	0.957

*FOGQ, Freezing of Gait Questionnaire; MDS, movement disorders society; SD, standard deviation; UPDRS, Unified Parkinson’s Disease Rating Scale.*

Data were collected from 24 participants in a laboratory setting designed to provoke FOG with a walking protocol, which included gait initiation, walking with 360- and 180-degree turns, walking in straight lines, passing narrow corridors, and walking through the crowded hospital halls between December 2016 and April 2017 (Mazilu et al., 2015a). Two nurses accompanied the participants during all the test procedures to prevent falls. The inertial measurement unit (IMU; BMI160, Bosch, Germany) generated nine signals sampled at 100 Hz as output. The nine signals represented the measurements of triaxial sensors: an accelerometer with sensitivity of 4,096 least significant bit (LSB)/g, a gyroscope with sensitivity of 16.4 LSB/deg/s, and a magnetometer. The data collection system contained seven wearable IMUs attached to different parts of the body (Figure 1C). Moreover, the participants underwent FOG evaluation with the sensors in all procedures. The entire experiment was recorded on video with an iPhone 6s Plus (A1699, Apple Inc., CA, United States), which was aligned with the signals on the timeline.

**FIGURE 2:**
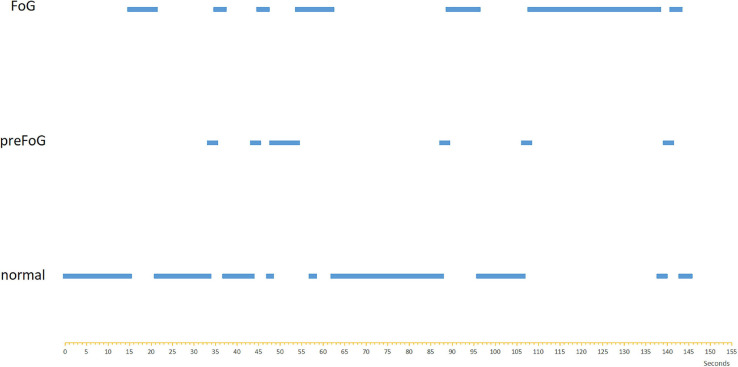
The event sequence of a video labeled with FOG, preFOG, and normal segments.

Currently, there is no detailed diagnostic criteria of FOG. To make it accurate, videos describing FOG in terms of the MDS-Sponsored Revision of the Unified Parkinson’s Disease Rating Scale (MDS-UPDRS) score were adopted. The FOG episodes were labeled offline by two independent gait experts who were blinded with respect to group allocation. If the labels of videos were inconsistent, the raters discussed labeling the gait as either FOG or not FOG. In total, 88 non-FOG episodes and 89 FOG episodes were captured. A FOG gait sequence in a patient video is shown as an example in Figure 2. The clinicians also labeled the start of other walking events, i.e., gait initiation, turns, and stops.

Twenty-four patients with FOG volunteered for this study. However, videos were excluded if the patient was blocked during recording and if the sensors fell off. Finally, 14 videos were included. The average inter-rater reliability was 0.928.

### Data Preparation

**FIGURE 3:**
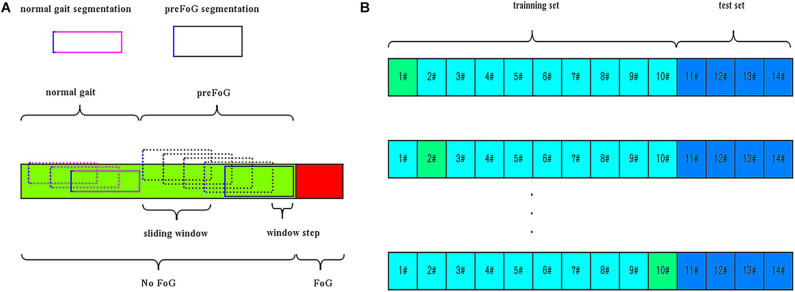
**(A)** Segmentation and pre-freezing of gait (preFOG) labeling. **(B)** Example of the training and evaluation data. In total, 10 patients’ data were used in training and 10-fold cross-validation, and four patients’ data were used in testing.

In addition to FOG and normal locomotion, the walking periods before FOG episodes were considered a third class called preFOG. It was hypothesized that there was a detectable deterioration of gait in this phase that precedes FOG (Mazilu et al., 2013). Different durations of the preFOG episodes were assumed. PreFOG episodes can be retrieved only through data mining from segments of data preceding FOG episodes, as shown in Figure 3A.

The accelerometer signals used in this study had burst outliers either larger than the 97.5th percentile or smaller than the 2.5th percentile. These outliers were replaced with the median value of the whole time series (Xia et al., 2018). The signals were also detrended by a high-pass filter.

The video and inertial signals were synchronized. The timestamps of the sensors were shown on the screen and were recorded in the first frame of the video. The signal data labeled with FOG were removed. To prepare the data instances for feature extraction, a sliding window was used to segment the whole time series into many overlapping data slices. The window size, which determines the length of the data segment, was fixed in advance in previous works. For the step size of the sliding window, it was evident that a smaller value of this parameter can generate more data instances. The signal data were segmented into slices by the means, as shown in Figure 3A.

According to statistical and digital signal processing knowledge and previous research (Zhang, 2017), the information on the raw signal features is listed in Table 2; in total, 924 (seven IMU sensors × 3D accelerometer and 3D gyroscope × 22 features) features were obtained (Pham et al., 2017). These features were then used to train a classification model. Due to the large difference in the scales of the data, the extracted feature data were standardized for model training.

**TABLE 2:** Information on the features extracted from the signal.

Expression	Remarks	Expression	Remarks
*F*_1_=*max*⁡(*x*)	Maximum value of the signal	$F_{12}=\frac{x_{m\,a\,x}}{\sqrt{\frac{1}{N_{s}}\,\sum_{i=1}^{N_{s}}x\,{\left(i\right)}^{2}}}$	Crest factor
*F*_2_=min(*x*)	Minimum value of the signal	$F_{13}=\frac{\bar{x}}{\sqrt{\frac{1}{N_{s}}\,\sum_{i=1}^{N_{s}}{\left(x\,\left(i\right)-\bar{x}\right)}^{2}}}$	Reciprocal coefficient of variation
$F_{3}=\frac{1}{N_{s}}\,\sum_{i=1}^{N_{s}}\left|x\,\left(i\right)\right|$	Mean of the absolute value of the signal	$F_{14}=\frac{1}{N_{s}}\,\sum_{j=1}^{N_{s}}{\left[\frac{x\,\left(j\right)}{\sqrt{\frac{1}{N_{s}}\,\sum_{i=1}^{N_{s}}{\left[x\,\left(i\right)-\bar{x}\right]}^{2}}}\right]}^{3}$	Skewness coefficient
*F*_4_=*max*⁡(*x*)−min(*x*)	Signal range	$F_{15}=\frac{1}{N_{s}}\,\sum_{j=1}^{N_{s}}{\left[\frac{x\,\left(j\right)}{\sqrt{\frac{1}{N_{s}}\,\sum_{i=1}^{N_{s}}{\left[x\,\left(i\right)-\bar{x}\right]}^{2}}}\right]}^{4}$	Kurtosis coefficient
$F_{5}=\sqrt{\frac{1}{N_{s}}\,\sum_{i=1}^{N_{s}}x\,{\left(i\right)}^{2}}$	Root mean square	$F_{16}=\frac{x_{m\,a\,x}}{\frac{1}{N_{s}}\,\sum_{i=1}^{N_{s}}x\,{\left(i\right)}^{2}}$	Clearance factor
$F_{6}=\frac{1}{N_{s}}\,\sum_{i=1}^{N_{s}}x\,\left(i\right)$	Mean of the signal	$F_{17}=\frac{x_{m\,a\,x}}{\frac{1}{N_{s}}\,\sum_{i=1}^{N_{s}}\left|x\,\left(i\right)\right|}$	Impulse factor
$F_{7}=\sqrt{\frac{1}{N_{s}}\,\sum_{i=1}^{N_{s}}{\left(x\,\left(i\right)-\bar{x}\right)}^{2}}$	Standard deviation	$F_{18}=\frac{\frac{1}{N_{s}}\,\sum_{i=1}^{N_{s}}{\left(\Delta \,x\,\left(i\right)-\Delta \,\bar{x}\right)}^{4}}{{\left[\frac{1}{N_{s}}\,\sum_{i=1}^{N_{s}}{\left(\Delta \,x\,\left(i\right)-\Delta \,\bar{x}\right)}^{2}\right]}^{2}}$	Energy operator $\Delta \,\bar{x}\,\frac{1}{N_{s}}\,\sum_{i=1}^{N_{s}}\Delta \,x\,\left(i\right)$ if the data point is not an endpoint, Δ*x* (*i*) *x* (*i*)^2^ − *x* (*i* + 1) × *x* (*i*−1) else, Δ*x* (*i*1) *x* (1)^2^ − *x* (2) × *x* (*N*_*s*_) Δ*x* (*N*_*s*_) *x* (*N*_*s*_)^2^ − *x* (1) × *x* (*N*_*s*_ − 1)
$F_{8}=\frac{\frac{1}{N_{s}}\,\sum_{i=1}^{N_{s}}{\left(x\,\left(i\right)-\bar{x}\right)}^{3}}{{\left[\sqrt{\frac{1}{N_{s}}\,\sum_{i=1}^{N_{s}}{\left(x\,\left(i\right)-\bar{x}\right)}^{2}}\right]}^{3}}$	Skewness depicts the symmetry of the signal distribution	$F_{19}=\frac{1}{N_{f\,f\,t}}\,\sum_{j=1}^{N_{f\,f\,t}}X\,\left(j\right)$	Mean frequency *X*(*j*): amplitude at the corresponding frequency
$F_{9}=\frac{\frac{1}{N_{s}}\,\sum_{i=1}^{N_{s}}{\left(x\,\left(i\right)-\bar{x}\right)}^{4}}{{\left[\sqrt{\frac{1}{N_{s}}\,\sum_{i=1}^{N_{s}}{\left(x\,\left(i\right)-\bar{x}\right)}^{2}}\right]}^{4}}$	Kurtosis depicts the steepness of the signal distribution	$F_{20}=\frac{\sum_{j=1}^{N_{f\,f\,t}}f\,\left(j\right)\times X\,\left(j\right)}{\sum_{j=1}^{N_{f\,f\,t}}X\,\left(j\right)}$	Center frequency *f*(*j*): frequency *X*(*j*): amplitude at the corresponding frequency
$F_{10}=\frac{1}{N_{s}}\,\sum_{i=1}^{N_{s}}{\left(x\,\left(i\right)-\bar{x}\right)}^{2}$	Variance of the signal	$F_{21}=\sqrt{\frac{\sum_{j=1}^{N_{f\,f\,t}}f\,{\left(j\right)}^{2}\times X\,\left(j\right)}{\sum_{j=1}^{N_{f\,f\,t}}X\,\left(j\right)}}$	Root mean square of the frequency *f*(*j*): frequency *X*(*j*): amplitude at the corresponding frequency
$F_{11}=\frac{\sqrt{\frac{1}{N_{s}}\,\sum_{i=1}^{N_{s}}{\left|x\,\left(i\right)\right|}^{2}}}{\frac{1}{N_{s}}\,\sum_{i=1}^{N_{s}}\left|x\,\left(i\right)\right|}$	Waveform factor	$F_{22}=\sqrt{\frac{\sum_{j=1}^{N_{f\,f\,t}}{\left(f\,\left(j\right)-f_{F\,C}\right)}^{2}\times X\,\left(j\right)}{\sum_{j=1}^{N_{f\,f\,t}}X\,\left(j\right)}}$	*f*(*j*): frequency *X*(*j*): amplitude at the corresponding frequency *f*_*FC*_: *F*_*20*_

***N_s_*, length of the signal; $\bar{x}$, mean of the signal; *x*_*max*_, maximum signal; *N*_*fft*_, length of the spectrum signal.**

### Experimental Setup

The window size, sliding step length, and preFOG duration involved in the extraction of features can be optimized to enhance the classifier’s performance. The window size, sliding step length, and preFOG duration were based on the sensor’s sampling rate. Each unit measurement means one sampling point, and the duration is 10 ms. The feature selection hyperparameters and the RF classifier were not the focus of this study. The Taguchi OED and RF were introduced into this study to build the models.

**FIGURE 4:**
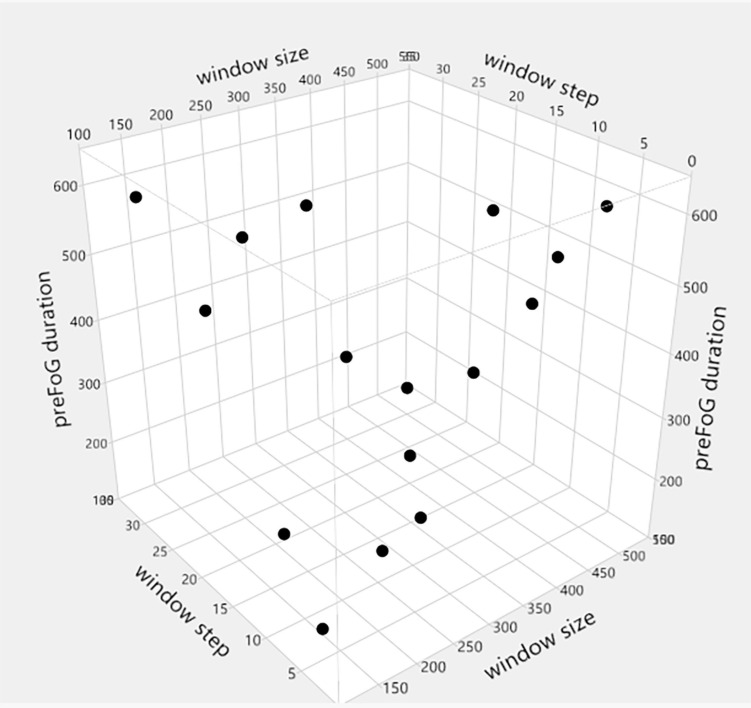
L_16_(4^3^) orthogonal experimental design (OED).

The OED is a type of general fractional factorial design (Cavazzuti, 2012). It is based on a design matrix proposed by Genichi Taguchi and allows the consideration of a selected subset of combinations of multiple factors at multiple levels. Taguchi orthogonal arrays (OAs) are balanced to ensure that all levels of all factors are considered equally. For this reason, the factors can be evaluated independently of each other despite the fractionality of the design. In the Taguchi OA design, only the main effects and two-factor interactions are considered, and higher-order interactions are assumed to be non-existent. In addition, designers are asked to identify (based on their knowledge of the subject matter) which interactions might be significant before conducting the experiment. The full factorial design of the three factors (window size, step length, and preFOG duration) with four levels consisted of 64 runs, while an L_16_(4^3^) OED scheme was chosen based on the OED. The different levels of window size, step length, and preFOG duration were chosen according to previous works. The details of the L_16_(4^3^) scheme are shown in Table 3 and visualized in Figure 4.

**TABLE 3:** Detailed orthogonal experimental design (OED) for optimizing the feature extraction parameters.

DOE name	ID	Window size	Step	PreFOG duration
T01	1	128	5	150
T02	2	128	10	250
T03	3	128	20	500
T04	4	128	30	600
T05	5	256	5	250
T06	6	256	10	150
T07	7	256	20	600
T08	8	256	30	500
T09	9	400	5	500
T10	10	400	10	600
T11	11	400	20	150
T12	12	400	30	250
T13	13	500	5	600
T14	14	500	10	500
T15	15	500	20	250
T16	16	500	30	150

*DOE, Design of experiment; preFOG, pre-freezing of gait.*

RF is an ensemble ML algorithm that combines a number of classification or regression trees and is based on the bagging technique. The RF algorithm is a powerful model and is used in many studies for the classification of FOG and other activity-related problems (Orphanidou et al., 2018). Common hyperparameters determine the accuracy of the RF classifier, such as the number of estimators (n_estimators), the maximum number or ratio of features (max_features), the maximum depth (max_depth), the minimum number of samples (min_samples_split), and the minimum number of samples needed by each leaf node (min_samples_leaf).

A set of parameters for feature extraction was fetched line by line from Table 3, and then the data were prepared according to the procedures shown in Figure 1B. The RF model was trained with the feature data and evaluated with the window-based strategy, and the evaluation results were stored in a table. The feature extraction parameters were optimized, and their main effects and interactions were analyzed.

After analysis, the optimized parameters were verified. The feature data were prepared according to the procedure described in the “Data Preparation” section with the optimized feature extraction parameters. A subset of features was selected to train the RF model according to the work by Zhang and Sawchuk (2011). The top-K method generated feature subsets, and the best subset was selected out according to the F1 score and kappa value. The prepared data were used to train the RF model, which was then evaluated with the episode-based strategy.

### Evaluation and Metrics

The preFOG prediction model was evaluated using leave-one-patient-out cross-validation, which meant a patient-independent evaluation. The data were split into a training set and a test set. The RF classifier was trained on feature data selected from N − 1 patients in the training set and evaluated with the data from the remaining patient, and some patients’ data were saved as the test set to avoid overfitting, as shown in Figure 3B. A window-based strategy was used for parameter optimization, and an episode-based strategy was used for verification of the optimized parameters.

The F1 score was adopted to evaluate the performance of the ML model with the setting configuration in the experiment. True positives were defined as gaits correctly classified by our method. False positives were defined as gaits that were identified as a certain class type but were found to be false from the video record. False negatives were gaits that were not identified as a certain class type, but the video record agreed with that class type. True negatives were those gaits for which both the applied method and the video agreed on the classification of not a certain class type (Djurić-Jovićić et al., 2013).

$$F_{1}-s\,c\,o\,r\,e=\frac{2\,T\,r\,u\,e\,P\,o\,s\,i\,t\,i\,v\,e\,s}{2\,T\,r\,u\,e\,P\,o\,s\,i\,t\,i\,v\,e\,s+F\,a\,l\,s\,e\,N\,e\,g\,a\,t\,i\,v\,e\,s+F\,a\,l\,s\,e\,P\,o\,s\,i\,t\,i\,v\,e\,s}$$

### Statistics Analysis

The Python (version 3.6.4) package scikit-learn (version 0.19.2) was used for the ML step. A trial version of JMP (version 13.2.0) and Minitab (version 18) were used to design the experimental scheme and to analyze the collected data.

The associated *p*-values of large effects are often very small. Visualizing these small values graphically can be challenging. When transformed to the LogWorth [−log10(*p*-value)] scale, highly significant *p*-values have large LogWorth values and non-significant *p*-values have small LogWorth values. A LogWorth of zero corresponds to a non-significant *p*-value of 1. Any LogWorth above two corresponds to a *p*-value less than 0.01.

Interaction effects occur when the effect of one variable depends on the value of another variable. Interaction effects are common in regression analysis, ANOVA, and designed experiments. In this paper, “window size^∗^preFOG duration” represents the interaction between the window size and preFOG duration.

## Results

**FIGURE 5:**
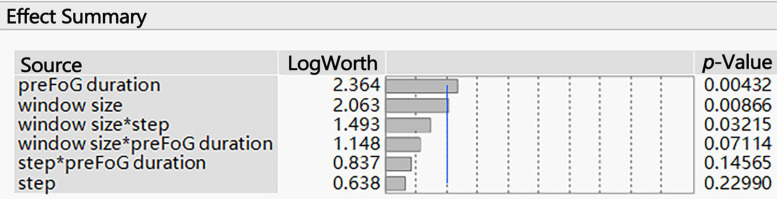
Summary of the effects of the feature extraction parameters on the kappa value. The p-values show the statistical significance of the association between the parameters and the kappa value. *Multiplication.

**FIGURE 6:**
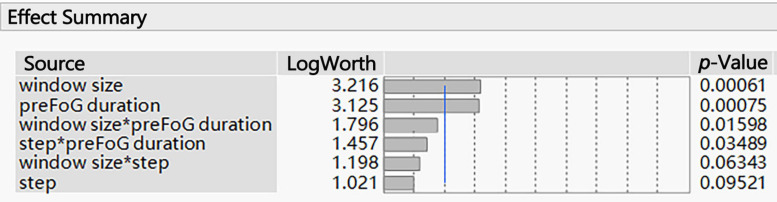
Summary of the effects of the feature extraction parameters on the F1 score. The *p*-values show the statistical significance of the association between the parameters and the F1 score. *Multiplication.

The parameters related to feature extraction included the window size, sliding step length, and preFOG duration. The preFOG duration (LogWorth: 2.364, *p*-value < 0.01) had the largest impact on the kappa value, followed by the window size (Figure 5). The window size (LogWorth: 3.216, *p*-value < 0.01) had the largest impact on the F1 score followed by the preFOG duration (Figure 6). Thus, there was a certain interaction between the window size and the preFOG duration defined in the experiment because of the statistically significant effect from the source item “window size^∗^preFOG duration” (Figure 6).

**FIGURE 7:**
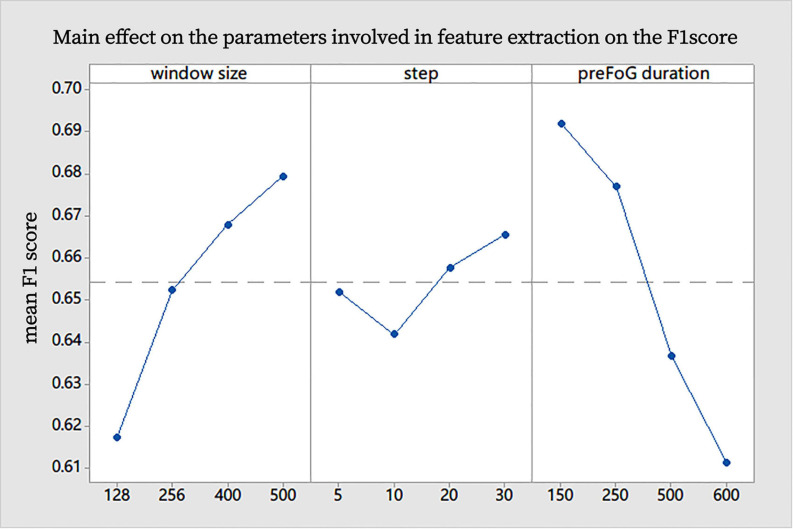
Main effect on the parameters involved in feature extraction on the F1 score.

Generally, the increase in the window size had an obvious positive effect on the F1 score, the increase in preFOG duration had an obvious negative effect on the F1 score, and the sliding step length did not affect our experiments (Figure 7). After analysis in the Minitab, the best combination of parameters in feature extraction was obtained and represented by a tuple (window size, step, and preFOG duration): (500, 20, and 250).

As the feature dimension is relatively high, this work also selects subsets of features and obtains the most important set of features {“F03,” “F05,” “F07,” “F09,” “F10,” “F11,” “F12,” “F13,” “F14,” “F15,” “F16,” and “F17”}. The OED was used to optimize the hyperparameters of the RF classifier. The optimal combination of parameters was presented as a tuple (n_estimators, max_features, max_depth, min_samples_split, and min_samples_leaf): (800, 0.1, 4, 4, and 4). In this tuple, max_features is the maximum ratio of features that can be used in a single subtree of the RF; “0.1” means a maximum of 924×0.1 = 92 features can be used.

With the optimized parameters, the RF model was trained and evaluated with an episode-based strategy. Under application conditions, FOG can be recorded as an episode, but normal gait cannot be evaluated as an episode. Therefore, we chose the false-positive rate, hit rate, and mean prediction time (MPT) for model evaluation. The false-positive rate, hit rate, and MPT were used as the indexes for evaluation of the prediction system. The false-positive rate increased with the hit rate. The false-positive rate, hit rate, and MPT were 27%, 68%, and 2.99 s, respectively.

## Discussion

Our key assumption was that FOG is not a sudden episode. In the period before FOG occurs, the patient’s kinematic features change. Based on this assumption, a prediction model was constructed, and the OED was used to optimize the hyperparameters to improve efficiency. To the best of our knowledge, this is the first time that the OED has been used for the ML-based prediction of FOG in PD. The interactions among the feature extraction parameters and the performance were first analyzed. The OED makes analyzing the effects of hyperparameters and finding the optimal ones more cost effective.

The OED was introduced to acquire optimized parameters involved in feature extraction. Having implemented the experimental design, the interactions were analyzed, the optimized parameters were obtained, and the efficiency of the test was improved. The existence of interactions hinted that it was necessary to tune the parameters simultaneously using combinatorial experiments and not a one-factor experimental design. Mikos et al. (2017) elucidated how the window size affects the MI between the feature and the FOG classification, which is a measure of the correlation between variables, and their works showed optimal window lengths for FOG classification vary across feature types. Mazilu et al. (2013) illustrated the effects of preFOG duration on the prediction and illuminated gait parameter changes prior to FOG (Ferster et al., 2015), but they still used the empirical and a *priori* preFOG duration. Sixteen runs from the OED were used to analyze the effects of parameters such as the window size, preFOG duration, and sliding window step length on the F1 score for preFOG identification, while the full factorial design of three factors with four levels required 64 runs. The workload was low, and the optimal parameters were obtained. The optimized preFOG duration and window size also implied the length of the preFOG period. With the improvement in the model training efficiency, more models can be trained with the same data scale and computing resources. Therefore, training personalized FOG prediction models for different individuals becomes possible. This is a possible direction to solve the problem of the poor generalizability of the model to different patients in real-world applications.

The classifier was evaluated by using a leave-one-patient-out, episode-based strategy and obtained an MPT of 2.99 s for FOG prediction with a false-positive rate of 27% and a hit rate of 68%. The performance of the model was not excellent in the patient-independent evaluation but was still comparable to previous works. As in the previous work by Mazilu et al. (2013) the FOG prediction performance was highly patient-dependent. The optimal parameters were not suitable for all patients, although our work solved these problems to some extent. We also developed a model with a patient-dependent method and obtained high performance with an F1 score of 0.89. In many ML models, the training and test data came from different patients, the identically distributed assumption is often violated, leading to poor performance. When the data from a patient were split into training and test data, the two datasets were often highly interdependent, leading to a good performance, but the model was overfit.

There were some limitations to our research. First, we did not include patients with a Hoehn–Yahr score greater than three since most of these patients have difficulty moving and are at risk of falling. The results might be affected if severe patients were included. Second, our method should be evaluated with the data of PD patients without FOG who were not included in our study. Third, the number of patients included in our study is small, weakening the experimental efficacy and supporting evidence. Future studies will be dedicated to improving the performance of the FOG prediction system by eliminating these defects.

Overall, a prediction classifier beneficial for early interventions for FOG was obtained. The OED was helpful in optimizing the hyperparameters. Larger-scale studies are needed.

## Data Availability Statement

The raw data supporting the conclusions of this article will be made available by the authors, without undue reservation.

## Ethics Statement

This study was approved by Ruijin Hospital affiliated to Shanghai Jiao Tong University School of Medicine. The patients/participants provided their written informed consent to participate in this study.

## Author Contributions

KR and SC designed and supervised the study, and double-checked the statistical analysis. ZC and GL collected the sample, performed the statistical analysis, and drafted the manuscript. All authors contributed to the article and approved the submitted version.

## Conflict of Interest

ZC, YL, XY, and KR were employed by the company Gyenno Science Co., Ltd. The remaining authors declare that the research was conducted in the absence of any commercial or financial relationships that could be construed as a potential conflict of interest.

## Abbreviations

ECG: electrocardiogram
FOG: freezing of gait
MPT: mean prediction time
MI: mutual information
ML: machine learning
OED: orthogonal experimental design
preFOG: prefreezing of gait
PD: Parkinson’s disease
RF: random forest
SCR: skin-conductance response.
